# Effects of naringenin supplementation in overweight/obese patients with non-alcoholic fatty liver disease: study protocol for a randomized double-blind clinical trial

**DOI:** 10.1186/s13063-021-05784-7

**Published:** 2021-11-13

**Authors:** Fatemeh Naeini, Zahra Namkhah, Helda Tutunchi, Seyed Mahdi Rezayat, Siavash Mansouri, Seyed Ali Jazayeri-Tehrani, Mehdi Yaseri, Mohammad Javad Hosseinzadeh-Attar

**Affiliations:** 1 Department of Clinical Nutrition, School of Nutritional Sciences and Dietetics, Tehran University of Medical Sciences, Tehran, Iran; 2 Endocrine Research Center, Tabriz University of Medical Sciences, Tabriz, Iran; 3 Department of Pharmacology, School of Medicine, Tehran University of Medical Sciences, Tehran, Iran; 4 National Iranian Oil Company (NIOC) Health and Family Research Center, Tehran, Iran; 5 Department of Epidemiology and Biostatistics, School of Public Health, Tehran University of Medical Sciences, Tehran, Iran

**Keywords:** Non-alcoholic-fatty liver disease, Naringenin, Metabolic parameters, Adiponectin, Neuregulin-4

## Abstract

**Introduction:**

Non-alcoholic fatty liver disease (NAFLD) is one of the main causes of chronic liver disease worldwide. Flavonoids, a group of natural compounds, have garnered a great deal of attention in the management of NAFLD because of their profitable effects on glucose and lipid metabolism, inflammation, and oxidative stress which are the pivotal pathophysiological pathways in NAFLD. Naringenin is a citrus-derived flavonoid with a broad spectrum of potential biological effects including anti-inflammatory and antioxidant properties, which may exert protective effects against NAFLD. The present clinical trial aims to examine the efficacy of naringenin supplementation on plasma adiponectin and neurogulin-4 (NRG-4) concentrations, metabolic parameters, and liver function indices in overweight/obese patients with NAFLD.

**Methods and analysis:**

This is a double-blind, randomized, placebo-controlled clinical study that will investigate the impacts of naringenin supplementation in overweight/obese patients with NAFLD. Liver ultrasonography will be applied to diagnose NAFLD. Forty-four eligible overweight/obese subjects with NAFLD will be selected and randomly assigned to receive naringenin capsules or identical placebo (each capsule contains 100 mg of naringenin or cellulose), twice daily for 4 weeks. Participants will be asked to remain on their usual diet and physical activity. Safety of naringenin supplementation was confirmed by the study pharmacist. The primary outcome of this study is changes in adiponectin circulating levels. The secondary outcomes include changes in NRG-4 levels, liver function indices, metabolic parameters, body weight, body mass index (BMI), waist circumference (WC), blood pressure, and hematological parameters. Statistical analysis will be conducted using the SPSS software (version 25), and *P* value less than 0.05 will be regarded as statistically significant.

**Discussion:**

We hypothesize that naringenin administration may be useful for treating NAFLD by modulating energy balance, glucose and lipid metabolism, oxidative stress, and inflammation through different mechanisms. The current trial will exhibit the effects of naringenin, whether negative or positive, on NAFLD status.

**Ethical aspects:**

The current trial received approval from the Medical Ethics Committee of Tehran University of Medical Sciences, Tehran, Iran (IR.TUMS.MEDICNE.REC.1399.439).

**Trial registration:**

Iranian Registry of Clinical Trials IRCT201311250155336N12. Registered on 6 June 2020

**Supplementary Information:**

The online version contains supplementary material available at 10.1186/s13063-021-05784-7.

## Introduction

Non-alcoholic fatty liver disease (NAFLD), defined as the accumulation of triglycerides within hepatocytes that exceeds 5% of liver weight, is an umbrella word for a broad spectrum of liver abnormalities including non-alcoholic steatohepatitis (NASH), advanced *fibrosis*, cirrhosis, and hepatocellular carcinoma (HCC) [1, 2]. Based on epidemiological evidence, the current global prevalence rate of NAFLD is 25% [3]. Different kinds of methods including platelet count, serum aminotransferase levels, the fibrosis-4 (FIB-4) index, NAFLD fibrosis score (NAS), imaging techniques, ultrasonography, and liver biopsy are used for the diagnosis of NAFLD. Despite the invasive nature of liver biopsy, it remains the gold standard to define and diagnose the severity of NAFLD [4]. However, ultrasonography can determine moderate and severe liver steatosis with an acceptable accuracy. A meta-analysis performed by Hernaez et al. [5] reported that sensitivity and specificity of ultrasonography were 84/8% and 93/6%, respectively. Experts are currently ongoing to rename NAFLD as metabolic-associated fatty liver disease (MAFLD) because of the close relationship between fatty liver and metabolic disorders [6]. Emerging evidence suggests that there is a bidirectional association between NAFLD and its risk factors including obesity, insulin resistance, dyslipidemia, oxidative stress, and inflammation [7]. Adipose tissue secretes a wide variety of bioactive substances, known as adipokines, which dysregulated secretion of these factors is involved in the development of NAFLD. Adiponectin and neuregulins are proposed to be linked with NAFLD [8]. Adiponectin, as an anti-inflammatory adipokine, is known to be inversely associated with insulin resistance, lipid accumulation, and NAFLD by playing a pivotal role in the modulation of hepatic glucose and lipid metabolism, stimulating the AMP-activated protein kinase (AMPK) signaling pathway, and decreasing the expression of peroxisome proliferator-activated receptor-α (PPAR-α) in the liver [9]. Regarding neuregulin-4 (NRG-4), a novel brown adipose tissue (BAT)-secreted adipokine, a growing body of evidence has demonstrated that it may protect metabolic hemostasis by regulating glucose and lipid metabolism. Also, hepatic de novo lipogenesis was controlled by BAT through NRG-4 [10]. In addition, NRG-4 transgenic mice had less lipid accumulation in the liver compared to the control mice [11].

It has become clear that diet, mainly dietary patterns, has a pivotal role in the progression of a wide range of liver conditions including NAFLD, NASH, fibrosis, and cirrhosis [12]. The most recent confirmed hypothesis explaining the pathogenesis of NAFLD is the multiple-hit theory which suggests multiple factors including insulin resistance, nutritional factors, gut microbiota, adipose tissue-secreted hormones, and genetic and epigenetic factors concurrently contribute to the induction of NAFLD in genetically predisposed individuals [13]. Although there are no approved pharmacological agents for the treatment of NAFLD, some dietary supplements including silymarin, omega-3, vitamin E, vitamin D, coenzyme Q10, and flavonoids can improve liver function indices in NAFLD patients [14]. Both animal and human studies have proposed that flavonoids exert protective activities against NAFLD by attenuation of nuclear factor kB (NF-kB) signaling, elevation of fatty acid oxidation in liver cells, and improvement of insulin sensitivity [15]. Among citrus-derived flavonoids, naringenin is a bitter flavanone which is mainly present in grapefruit and possesses incredible biological properties including antiviral, antioxidant, anti-inflammatory, anti-cancer, anti-hyperlipidemic, anti-hyperglycemic, and weight-lowering activities [16]. Since NAFLD is strongly related to obesity, metabolic parameters, inflammation, and oxidative stress, it seems that naringenin administration would lead to an improvement of liver indices in NAFLD patients through modulating energy hemostasis, increasing fatty acid oxidation, regulating glucose metabolism, inhibiting the expression of pro-inflammatory and pro-fibrotic signaling pathways, and attenuating oxidative stress [17]. It should be noted that consumption of most of the flavonoids like naringenin caused no side effects due to their low bioavailability and rapid metabolism and elimination [18–20]. As far as we know, no clinical trial has evaluated the effects of naringenin in NAFLD patients, yet. A recent study performed by Ahmed et al. [21] found that administration of naringenin (20 mg/kg for 4 weeks) resulted in a significant reduction in aminotransferase, total bilirubin, and levels of inflammatory biomarkers; downregulation of hepatic proapoptotic proteins; and upregulation of hepatic anti-apoptotic proteins in Wistar rats with liver injury. Moreover, an in vivo study by Chen et al. [22] concluded that naringenin and nano-naringenin administration (100 mg/kg and 25 mg/kg for 1 week, respectively) reduced serum aminotransferase levels and lipid accumulation in the liver of mice fed methionine choline-deficient diet. Furthermore, another study investigating the effects of naringenin on isoniazid- and rifampicin-induced apoptosis in hepatic injury demonstrated an improvement in anti-apoptotic effects, liver indices, and liver antioxidant content by naringenin supplementation (100 mg/kg for 2 weeks) [23]. The present clinical study aims to assess the effects of naringenin administration on plasma adiponectin concentrations in overweight/obese patients with NAFLD as the primary objective. Moreover, secondary objectives of this research include evaluating alterations in plasma NRG-4 concentrations, metabolic parameters, indices of liver function, body weight, BMI, WC, blood pressure, and hematological parameters.

## Methods and analysis

### Study design

**Fig. 1:**
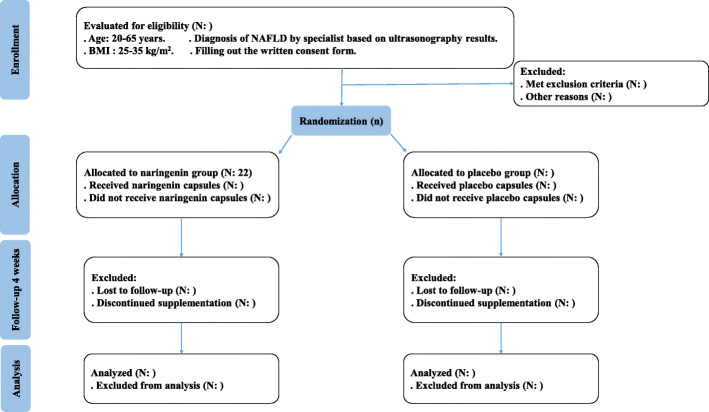
Study flowchart. BMI, body mass index; NAFLD, non-alcoholic fatty liver disease

The present research, a placebo-controlled, randomized, double-blind clinical trial examining the impacts of naringenin administration in overweight/obese NAFLD patients, is registered at the Iranian Registry of Clinical Trials (ID: IRCT20131125015536N12). This trial will be performed at the National Iranian Oil Company (NIOC) central hospital, Tehran, Iran. Written informed consent will be obtained from participants before participation in the research project by researchers. We developed the study protocol based on the Standard Protocol Items: Recommendations for Interventional Trials (SPIRIT) 2013 checklist (supplemental file 1). The timeline of the trial and study flow chart of enrollment, allocation, intervention, and assessment are presented in Table 1 and Fig. 1, respectively. Any amendments of the present study protocol which are related to safety and benefit of patients and the protocol deviations and unintentional alterations in study protocol that do not affect subject’s rights, study’s risk or benefit, the integrity of data, and safety or welfare, will require to be confirmed by the Department of Clinical Nutrition and Medical Ethics Committee of Tehran University of Medical Sciences, Tehran, Iran, before the study conduction. Any alteration in the study protocol will be sent to the *Trials* journal (www.trialsjournal.biomedcentral.com).

**Table 1:** Timeline of the trial

Explanation of the trial activities	Time (months)															
	1	2	3	4	5	6	7	8	9	10	11	12	13	14	15	16
Material preparation	*	*	*	*												
Recruitment					*	*	*	*								
Clinical assessments at baseline					*	*	*	*								
Nutritional assessments at baseline					*	*	*	*								
Biochemical assessments at baseline					*	*	*	*								
Hepatic steatosis and fibrosis assessments at baseline					*	*	*	*								
Intervention									*	*	*	*	*			
Clinical assessments after intervention									*	*	*	*	*			
Nutritional assessments after intervention									*	*	*	*	*			
Biochemical assessments after intervention									*	*	*	*	*			
Hepatic steatosis and fibrosis assessments after intervention									*	*	*	*	*			
Data analysis														*	*	
Writing the final report of the trial																*
The expected time	*	*	*	*	*	*	*	*	*	*	*	*	*	*	*	*

### Study setting

The current research will be performed at the NIOC central hospital, Tehran, Iran, and participants will be selected from NAFLD patients who are newly diagnosed by an expert radiologist using ultrasonography.

### Study participants and enrollment

NAFLD patients will be recruited from the clients of the NIOC central hospital ultrasound department by two independent researchers. It should be noted that due to the simultaneous coronavirus disease 2019 (COVID-19) pandemic and the implementation of the current study, researchers should expand their presence time in NIOC central hospital ultrasound department for achieving adequate participant enrollment to reach the target sample size. An adept radiologist with sufficient expertise in the diagnosis of fatty liver through ultrasonography will diagnose patients with NAFLD. Patients that satisfy the following inclusion criteria will be eligible to participate in this research project.

#### Inclusion criteria

The inclusion criteria are considered as follows: individuals aged between 20 and 65 years, subjects whose BMI ranged between 25 and 35 kg/m^2^, diagnosis of NAFLD by a specialist based on ultrasonography results, and patients who fill out a written informed consent for participation in this study. To ensure minimal loss to follow-up, we will include patients who live in Tehran.

#### Exclusion criteria

The exclusion criteria will include subjects who are pregnant or breastfeeding or menopause; who are afflicted by diabetes mellitus, hypertension, human immunodeficiency virus, gastrointestinal diseases, organs failure, thyroid abnormalities, kidney disorders, autoimmune diseases, severe mental illnesses, and different kinds of malignancies; patients who had cardiovascular accidents in the last 3 months; professional athlete; and individuals who are addicted to drugs and alcohol. In addition, subjects who regularly consumed herbal essences such as silymarin, antioxidants, omega-3, probiotics, multivitamin-mineral supplements, NSAIDS, antibiotics, corticosteroids, anti-hypertension, anti-hyperglycemic, and weight-lowering agents in the last 3 months will be excluded. We will also exclude patients with pathological liver conditions including cirrhosis, virus-related hepatitis, and liver transplantation. To ensure minimal loss to follow-up, we will exclude participants who do not live in Tehran. Also, patients who are unwilling to continue collaborating on the research and have poor compliance (less than 90%) with the assigned intervention will be withdrawn from follow-up.

### Randomization and blinding

Stratified permuted block randomization will be applied to stratify participants into different stratum and blocks (block sizes 4, 4, 2, 6, 2) based on gender and probable confounders including age, BMI, and NAFLD severity. Each block will be randomly allocated to the intervention or control groups. The sequence of the blocks will be prepared for each stratum using a random number table. For each patient in a definite block, a matched person in terms of the aforementioned variables would be considered in that block. Participants and investigators will be blind to the trial group assignments until the end of the study and data analysis, with the exception of the study pharmacist. A randomization list and sequentially numbered drug containers will be provided by the study pharmacist.

### Study interventions

Forty-four eligible NAFLD patients will be randomly allocated to placebo (*n* = 22) and intervention groups (*n* = 22). Naringenin capsules (contain 100 mg naringenin) and identical placebo capsules (contain 100 mg cellulose) will be respectively administered to patients in the intervention and control groups twice a day, before lunch and dinner, for 4 weeks. Naringenin and placebo capsules will be similar in weight, size, shape, taste, color, odor, and lot number. Participants will be informed how to use their supplements, and they will be followed by phone calls once a week. Also, due to ethical considerations, physical activity and dietary recommendations will be provided by the researchers in order to improve participants’ lifestyles in both naringenin and placebo groups. No specialized diet or physical activity plan will be provided for study patients.

### Outcomes

#### Primary and secondary outcomes

The primary outcome of the current research includes alteration in plasma adiponectin concentrations. Alterations in plasma NRG-4 concentrations; lipid profile including triglyceride (TG), high-density lipoprotein cholesterol (HDL-C), low-density lipoprotein cholesterol (LDL-C), and total cholesterol (TC); glycemic parameters including fasting blood sugar (FBS), fasting blood insulin (FBI), hemostatic model assessment of insulin resistance (HOMA-IR), and Quantitative Insulin Sensitivity Check Index (QUICKI); levels of aminotransferases including aspartate aminotransferase (AST) and alanine aminotransferase (ALT); liver steatosis; NFS; weight; BMI; WC; blood pressure; hematological parameters; energy intake; and consumption of energy-contributing nutrients including carbohydrates, fat, and protein are considered as secondary outcomes.

### Measurements and assessments

#### Clinical assessments

Medical history, alcohol consumption, smoking history, and demographic factors including age, sex, race, income level, employment status, education level, marital status, and homeownership will be asked from all the patients at the beginning of the research. Blood pressure will be measured using a mercury sphygmomanometer after at least 5-min resting. The measurement will be performed on two occasions, and the mean of the two will be considered as the individual’s blood pressure.

#### Assessment of physical activity

For estimating the physical activity level of participants, an International Physical Activity Questionnaire-Short Form (IPAQ-SF) which was previously validated in Iran will be applied. The IPAQ form computes and reports physical activity in metabolic equivalents per minute (MET-min) per week. The levels of physical activity will be reported in three categories including low activity, moderate activity, and high activity levels based on the IPAQ scoring protocol [24, 25]. To control confounders, alterations in physical activity levels throughout the study will be taken into consideration for statistical analysis.

#### Nutritional assessments

At the onset and end of the trial, body weight and height will be measured to the nearest 0.1 kg and 0.1 cm using the Seca scale and stadiometer (Seca), respectively. The individuals will be measured when they are barefoot and wearing light clothing. BMI is computed by dividing weight in kilograms by height in meters squared. WC is measured using the middle of the lowest gear, the high point of the iliac crest, and on the biggest environmental gluteal muscle, respectively. A 3-day food record (2 weekdays and 1 weekend day), a valid instrument for the evaluation of diet, will be used for dietary assessment in patients. The 3-day food record will be completed at the beginning and end of the intervention by face-to-face interview. We will analyze the information about dietary consumption by the Nutritionist IV software. To control confounders, alterations in dietary intakes throughout the study will be taken into consideration for statistical analysis.

#### Biochemical measurements

Blood samples (10 ml) will be drawn following a 12-h overnight fasting and centrifuge at 3000 rpm for 5 min to extract serum samples pre- and post-intervention. Complete blood count and serum albumin will be assessed from the whole blood that was placed in EDTA tubes using Hematology Analyzer (Nihon Kohden Celltac alpha MEK-6510) and chromatography method, respectively. The concentrations of TC, TG, LDL-C, HDL-C, and liver transaminases including AST and ALT will be evaluated instantly on fresh blood samples using commercial kits (Pars Azmoon Inc. kit, Tehran, Iran) before and after the intervention. The concentrations of FBI and FBS will be measured using commercial enzyme-linked immunosorbent assay (ELISA) kit (IBT, USA) and glucose oxidase method, respectively. Serum adiponectin and NRG-4 levels will be assessed using ELISA kits (Human Adiponectin and NRG-4 ELISA kits, Crystal Day Bio-Tec, Shanghai, China) pre- and post-intervention. The following formulas will be used to determine QUICKI and HOMA-IR indexes.

$$ \mathrm{QUICKI}:1/\left(\log\ \left(\mathrm{fasting}\ \mathrm{insulin}\ \upmu \mathrm{U}/\mathrm{ml}\right)+\log\ \left(\mathrm{fasting}\ \mathrm{glucose}\ \mathrm{mg}/\mathrm{dl}\right)\right) $$

$$ \mathrm{HOMA}-\mathrm{IR}:\left[\mathrm{fasting}\ \mathrm{insulin}\ \left(\upmu \mathrm{IU}/\mathrm{ml}\right)\times \mathrm{fasting}\ \mathrm{glucose}\ \left(\mathrm{mg}/\mathrm{dl}\right)\right]/405 $$

#### Hepatic steatosis and fibrosis assessment

The severity of fatty liver will be evaluated using ultrasonography by an expert radiologist. The presence of fatty liver will be determined in a qualitative manner based on accepted criteria including a diffuse hyper-echoic texture as mild, increased liver echotexture compared to the renal cortex as moderate, and vascular blurring and deep attenuation as severe. Also, the following formulas will be used to determine NAS and FIB-4 indexes. Regarding the NAS index, patients will be classified as low, intermediate, or high probability of fibrosis according to a score < − 1.445, − 1.445 ≤ score ≤ 0.676, and score > 0.676, respectively [26]. Regarding the FIB-4 index, a value of < 1.30 will be considered as a low probability of fibrosis, a value of > 2.67 will be considered as a high probability of fibrosis, and values between 1.30 and 2.67 will be considered as an indeterminate probability of fibrosis [27].

$$ \mathrm{NAS}:-1.675+0.037\times \mathrm{age}\ \left(\mathrm{years}\right)+0.094\times \mathrm{body}\ \mathrm{mass}\ \mathrm{index}\ \left(\mathrm{kg}/{\mathrm{m}}^2\right)+1.13\times \mathrm{impaired}\ \mathrm{fasting}\ \mathrm{glucose}/\mathrm{diabetes}\ \left(\mathrm{yes}=1,\mathrm{no}=0\right)+0.99\times \mathrm{AST}/\mathrm{ALT}\ \mathrm{ratio}-0.013\times \mathrm{platelet}\ \left(\times {10}^9/\mathrm{l}\right)-0.66\times \mathrm{albumin}\ \left(\mathrm{g}/\mathrm{dl}\right) $$

$$ \mathrm{FIB}-4:\mathrm{age}\ \left(\mathrm{years}\right)\times \mathrm{AST}\ \left(\mathrm{IU}/\mathrm{l}\right)/\left(\mathrm{platelet}\ \mathrm{count}\ \left({10}^9/\mathrm{l}\right)\times \surd \mathrm{ALT}\ \left(\mathrm{IU}/\mathrm{l}\right)\right) $$

### Sample size calculation

By consideration of a type I error of 5% (*α* = 0.05), a power of 90%, assumptions in the control group of 0.99, and the expected treatment effects of 0.93, according to the mean plasma levels of adiponectin as a primary outcome [28], the sample size was computed to be 21 for each group based on the two-sided *t* test. To compensate for an approximate drop-out rate of 5% during the study period, we elevate the final sample size to 22 subjects in each group.

### Safety evaluation

Based on the study performed by Chen et al. [22] in which the administration of 100 mg/kg naringenin significantly decreased the serum levels of liver enzymes in mice fed methionine choline-deficient diet and the following formula for dose conversion between animals and human [29], the naringenin dosage was determined as 200 mg/day by study pharmacist.

$$ \mathrm{Human}\ \mathrm{equivalent}\ \mathrm{dosage}\ \left(\mathrm{mg}/\mathrm{kg}\right)=\mathrm{animal}\ \mathrm{dosage}\ \left(\mathrm{mg}/\mathrm{kg}\right)\times \left({Km}_{\mathrm{animal}}/{Km}_{\mathrm{Human}}\right) $$

We will call the patients weekly and ask them about occurring any adverse events following naringenin consumption. If the reported adverse events are correlated with the naringenin consumption, participants will be asked to stop taking naringenin supplements, and also, they will be immediately referred to a specialist for therapy.

### Data management and monitoring

A clinical trial monitor occasionally supervises the study progress; ensures patient rights and well-being are safeguarded; the protocol, ethical requirements, standards, and regulations are being followed; the essential documentation is available; and collected data are accurate as there were recorded. One of the investigators will check the coding, security, and storage of the data. In addition, he/she will evaluate the data entry and data values twice. If any participant reports the occurrence of adverse events, more information is required to make a decision about excluding the participants from the trial. Unblinding is permissible in this situation based on the Medical Ethics Committee criteria.

### Adherence to the intervention

The study progress will be pursued by calling the patients once a week to ensure that they regularly consume the capsules. Adherence to the intervention will be checked by counting the returned capsules at the half and end of the trial visits. The compliance rate will be computed according to the following formula, and poor compliance will be considered as less than 90% [30].

$$ \mathrm{Compliance}\ \mathrm{rate}:\left(\mathrm{capsules}\ \mathrm{taken}/\mathrm{capsules}\ \mathrm{prescribed}\right)\times 100 $$

### Statistical analysis

Statistical analysis will be conducted using the SPSS software (version 25, SPSS Inc., and Chicago, IL, USA). The Kolmogorov-Smirnov test will be applied to examine the normality of data. Moreover, we will use the chi-square test and Fisher’s exact test to compare the categorical variables. Also, the independent sample *t* test and Wilcoxon rank-sum test will be applied to compare the continuous variables within the groups, whereas we will use the paired sample *t* test and Mann-Whitney *U* test for between-group comparisons. Normally distributed variables will be reported as the mean and standard deviation, while the median and interquartile range (IQR) will be used for reporting non-normally distributed variables. To compare the differences in primary and secondary outcomes between the two study groups at the end of the trial and also adjust the final findings for potential confounders, we will apply the analysis of covariance (ANCOVA) test. *P* value less than 0.05 will be regarded as statistically significant.

## Discussion

NAFLD is the most common cause of liver dysfunction in the world [31]. Global policies are needed to address the disability burden of NAFLD and other chronic liver diseases [32]. NAFLD usually did progress to advanced fibrosis and cirrhosis in patients who are not treated properly [33]. Lifestyle modifications including regular exercise and dietary interventions are the main topics of concern for the management of NAFLD [34].

Naringenin is a colorless trihydroxyflavonone which belongs to the 4′-hydroxyflavanone group. Similar to the other flavonoids, naringenin exerts multiple favorable effects on human health [35]. A large number of experimental studies concluded that naringenin has weight-lowering properties and may improve obesity-related NAFLD through modulation of energy hemostasis and prevention of adipogenesis [36–39]. Furthermore, a growing body of evidence from animal studies found that naringenin treatment resulted in an improvement in hepatic steatosis and metabolic parameters by reducing fasting plasma glucose, fasting plasma insulin, TG and TC levels in liver and plasma, and apoB-100 secretion [38, 40–43]. Moreover, initial clinical studies reported that administration of naringenin contributed to the protection against oxidative stress and inflammation related to NAFLD through decreasing liver lipid peroxidation, increasing liver antioxidant content, and attenuating hepatic pro-inflammatory gene expression including tumor necrosis factor-α (TNF-α), interleukin-6 (IL-6), and IL-1β via inhibition of NF-kB activation [21, 44–46]. Although many experimental studies have investigated the effects of naringenin on NAFLD and its related risk factors, there is no clinical trial assessing the efficacy of naringenin in patients with NAFLD. This gap can be addressed through the conduction of relevant clinical trials with human subjects.

The pathogenesis of NAFLD, a clinical syndrome spans from simple steatosis to cirrhosis, includes insulin resistance, lipotoxicity, hepatic inflammation, and obesity [47]. Regarding the NAFLD pathophysiology, we hypothesize that naringenin may improve liver function indices in overweight/obese patients with NAFLD by modulating lipid and glucose metabolism, lowering weight and adiposity, and decreasing hepatic oxidative stress and inflammation. According to the aforementioned hypothesis, the present trial is designed. Suggesting a natural component as an option for the management and treatment of NAFLD is one of the novel characteristics of the current clinical study. In addition, this research may lead to the cost-lowering effects due to the high burden and prevalence of NAFLD in Iran and worldwide.

## Trial status

The recruitment started on 18 January 2021 and will be almost finished on 20 May 2021.

## Supplementary Information

**Additional file 1.**

## Data Availability

The first and corresponding authors will have access to the interim results and make the final decision to terminate the trial. The non-identifiable individual patients’ data will become available to other researchers in academic institutions.

## Abbreviations

ALT: Alanine aminotransferase
AMPK: AMP-activated protein kinase
AST: Aspartate aminotransferase
BAT: Brown adipose tissue
BMI: Body mass index
COVID-19: Coronavirus disease 2019
ELISA: Enzyme-linked immunosorbent assay
FBI: Fasting blood insulin
FBS: Fasting blood sugar
FIB-4: Fibrosis-4
Hb: Hemoglobin
HDL-C: High-density lipoprotein cholesterol
HOMA-IR: Hemostatic model assessment of insulin resistance
IL: Interlukin
IPAQ-SF: International Physical Activity Questionnaire-Short Form
LDL-C: Low-density lipoprotein cholesterol
MAFLD: Metabolic-associated fatty liver disease
NAFLD: Non-alcoholic fatty liver disease
NASH: Non-alcoholic steatohepatitis
NF-kB: Nuclear factor kB
NFS: NAFLD fibrosis score
NIOC: National Iranian Oil Company
NRG-4: Neurogulin-4
PPAR: Peroxisome proliferator-activated receptors
QUICKI: Quantitative Insulin Sensitivity Check Index
TC: Total cholesterol
TG: Triglyceride
TNF-α: Tumor necrosis factor-α
WC: Waist circumference
